# Implementation of exclusive enteral nutrition in pediatric patients with Crohn’s disease—results of a survey of CEDATA-GPGE reporting centers

**DOI:** 10.1186/s40348-022-00139-x

**Published:** 2022-04-05

**Authors:** Sarah Peters, Serdar Cantez, Jan De Laffolie, CEDATA Study Group

**Affiliations:** 1 Institut für Ernährungswissenschaft, Justus-Liebig-Universität, Giessen, Germany; 2 Department for General Pediatrics & Neonatology, University Hospital Giessen, Giessen, Germany

**Keywords:** Inflammatory bowel disease, Crohn’s disease, Exclusive enteral nutrition, Patient registry, Pediatric

## Abstract

**Background:**

Exclusive enteral nutrition (EEN) is the first-line therapy for pediatric-onset Crohn’s disease (CD) patients. CEDATA-GPGE® is the largest patient registry for children and adolescents with inflammatory bowel disease (IBD) in Europe, collecting data from over 5000 patients since 2004 in Germany and Austria. Since the application of EEN over 8 weeks is difficult and a high dropout rate is often described, the mode of application including a supporting structure is crucial for success. The aim of this study was to ascertain the variation in the application of EEN across the participating centers and to associate these with the outcome.

**Results:**

Thirty-one centers responded to the survey (81.6%). 88.5% of CD patients were recommended EEN for induction therapy, 71.8% actually started with EEN, and 22.1% terminated the EEN prematurely. The duration of EEN typically lasted 6 to 8 weeks, and the polymeric formula was mainly used. 80.6% of the clinics added flavorings to the formulas. After EEN, the most preferred diet for maintenance therapy was a healthy, well-balanced diet considering individual intolerances.

**Conclusions:**

EEN is widely recommended as an induction therapy by the German and Austrian pediatric gastroenterologists for children and adolescents with CD. However, this questionnaire-based study has shown a wide variation in EEN protocols used by the different pediatric clinics of CEDATA-GPGE®.

**Supplementary Information:**

The online version contains supplementary material available at 10.1186/s40348-022-00139-x.

## Background

Inflammatory bowel diseases (IBD) are on the rise around the globe including pediatric-onset IBD [1]. Approximately every fourth patient receives the diagnosis before the age of 18 [2]. The role of nutrition in the pathogenesis of disease and inflammatory activity is still widely discussed. Findings from animal and human studies indicate the pathogenic character of plenty of animal fat and protein, sugar, and convenience food. Fruits, vegetables, and fibers are associated with a protective effect [3–5]. There is an increasing demand and rising body of evidence for nutritional interventions as therapy.

Exclusive enteral nutrition (EEN) over 6 to 8 weeks is the current first-line therapy for induction of remission for children and adolescents with luminal Crohn’s disease (CD) [6, 7]. However, the treatment protocols for EEN vary among countries [8–11].

It became clear in early reviews (2000er) that the mode of delivery and support of patients is crucial for adherence to an effective therapy. There is still insufficient data on dietary intervention maintenance therapy.

In Germany and Austria pediatric IBD patients (0–18 years of age) are included in the registry CEDATA-GPGE® of the Society for Pediatric Gastroenterology and Nutrition (GPGE) [12].

The aim of the present study was to determine how EEN was administered to pediatric patients in the hospitals of CEDATA-GPGE®.

## Methods

### Participants and questionnaire

A 25-item questionnaire (Additional file 1) was developed for this study and sent by mail and e-mail to the 38 pediatric gastroenterology units of CEDATA-GPGE® who were actively contributing patient data at that time. The survey was divided into two parts: part one was created for physicians and part two for dieticians. The physicians were asked to complete part one of the survey and to subsequently pass on the questionnaire to their dieticians to fill out part two. In case a dietician was not involved or employed, they were asked to complete the entire questionnaire.

Initial questions in part one asked for the number of pediatric patients with CD and for details of the current EEN protocol at their centers. This included the duration of EEN, the type of formula used and how many patients got recommended, started, and quit an EEN completely. Part two asked for the dosage of EEN, added flavorings used, and the protocol for reintroduction of foods and beverages at the end of EEN. A second and third e-mail was sent after 4 and 8 weeks, respectively, to remind those who had not yet responded.

The age structure of the pediatric CD patients was collected from CEDATA-GPGE®. Due to the lack of recording the partial enteral nutrition (PEN) and the dropout rate of EEN in CEDATA-GPGE®, both are based on the subjective assessment of the physician.

### Statistical analysis

Descriptive statistics and exploratory analysis were performed using IBM SPSS Statistics version 26. Response values for each question were tabulated as frequency counts and were reported as percentages of valid responses for a given question. Results were expressed in frequencies (percentage and number = *N*) and median (mdn), standard deviation (SD), interquartile range (IQR), and minimum (min) and maximum (max).

Mann-Whitney *U* tests were used to examine the differences between two independent samples. Spearman’s and Pearson’s correlation was used to evaluate correlations between two variables. *P* values less than 0.05 were considered statistically significant.

Due to the low power and the smallness of the sample, the standardized effect *r* size was calculated. The Cohen classification was used to assess the size of the effect: *r*_*s*_ = .10 ≙ weak effect, *r*_*s*_ = .30 ≙ medium effect, and *r*_*s*_ = .50 ≙ strong effect.

## Results

### Background of respondents

Thirty-three out of 38 pediatric gastroenterology centers of CEDATA-GPGE®, replied to the survey. Two of them had to be excluded from the study because they did not treat patients with CD, but instead referred them to other hospitals. In total, 31 CEDATA-GPGE® centers were included (81.6% of those asked).

Forty-eight percent of the returned surveys were completed by both physicians and dieticians. The other half of the questionnaire was answered by the gastroenterologists alone since they did not employ or involve dieticians, specialized in EEN.

A mean of 57 patients was looked after by each hospital of CEDATA-GPGE® (SD = 54.1, mdn = 40). The average age of patients with CD at time of diagnosis was 11.8 years (SD = 3.1, mdn = 12, IQR = 10–14, *N* = 351).

### Use and protocols of EEN

In 93.5% (29/33) of the clinics surveyed, EEN began during an inpatient stay. In 12.9% (4/33), the patient was informed about EEN on an outpatient basis. Two clinics replied that they do it both ways.

The administration of EEN typically lasted 6 to 8 weeks (96.8%, 30/31). One clinic replied that EEN administration lasted over 8 weeks. Reported reasons for this variation in duration of EEN were the noncompliance of patients or imminent termination of the intervention (50%, 7/14) or the lack of therapy success (35.7%, 5/14). Other reported reasons were extreme dystrophy and a repetition of EEN.

Overall, the hospitals recommended 88.5% of the pediatric CD patients an EEN for induction therapy (SD = 16.5, *N* = 27). 71.8% of the children and adolescents began an EEN (SD = 18.1, *N* = 27) and 77.9% completed this intervention (SD = 12.1, *N* = 30). The percentages are estimated values of the pediatric gastroenterologists, and concrete patient numbers were not requested.

**Fig. 1:**
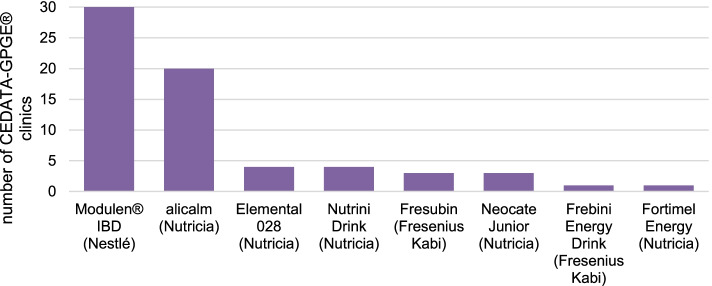
Formula used in the clinics of CEDATA-GPGE®; *N* = 30. EEN, exclusive enteral nutrition (own illustration)

Polymeric formulas were used by most of the centers. In total, eight different formulas were utilized across the centers surveyed, with Modulen IBD® (Nestlé Health Science, Frankfurt, Germany) (48.4%, 30/30) and alicalm® (Nutricia, Frankfurt, Germany) (32.2%, 20/30) being the most frequently used. 19.4% (12/30) used other formulas (Fig. 1).

**Fig. 2:**
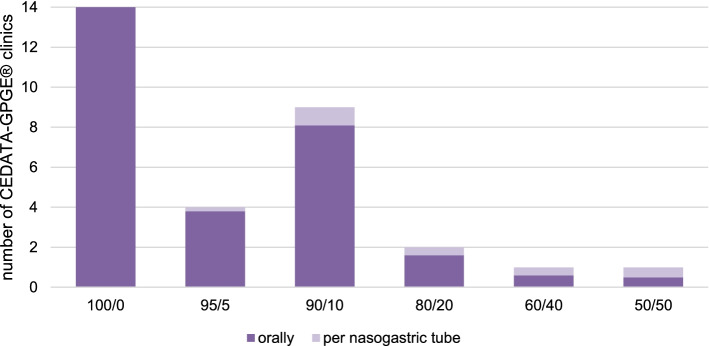
Application of formula during EEN (orally/per nasogastric tube) in the clinics of CEDATA-GPGE®; *N* = 31; in %. EEN, exclusive enteral nutrition (own illustration)

Most respondents reported that they preferred to administer the formula orally to their patients: 45.2% (14/31) of CEDATA-GPGE® clinics treated the CD patients exclusively with orally administered formula during EEN and 29.0% (9/31) of the centers reported that 90% of the patients tolerated the formula orally and 10% required administration via a tube (Fig. 2). The percentages are the estimated values for the number of patients treated by the pediatric gastroenterologists, and specific patient numbers were not requested.

**Fig. 3:**
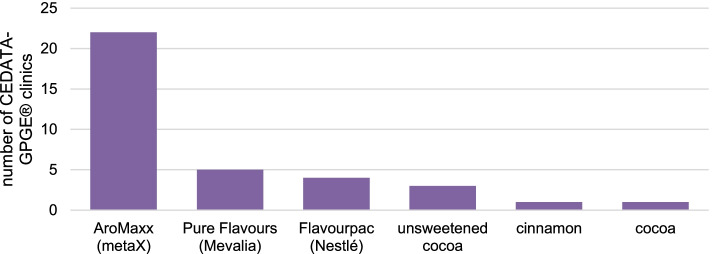
The addition of flavorings to the formula in EEN in the clinics of CEDATA-GPGE®; *N* = 31. EEN, exclusive enteral nutrition (own illustration)

The addition of flavorings to formula was permitted in 80.6% (25/31) of centers. These included commercially available formula flavorings: 71.0% (22/31) AroMaxx® (metaX, Friedberg, Germany), 16.1% (5/31) Pure Flavours (Dr. Schär, Postal, Italy), and 12.9% (4/31) Flavourpac® (Nestlé Health Science, Frankfurt, Germany). Other reported flavorings included cocoa (9.7%, 3/31), cinnamon, and unsweetened cocoa (3.1%, 1/31) (Fig. 3).

During the period of EEN, most centers (61.3%, 19/31) allowed fluids (still mineral water, unsweetened fruit, or herbal teas) and sugar-free chewing gum. In exceptional cases, 35.5% (11/31) permitted clear soup, foods of the Crohn’s Disease Exclusion Diet (CDED) [13], and diluted apple juice. Reported exceptions were made due to imminent termination of EEN despite clinical improvements or during the repetition of EEN.

### Dropout rates of EEN

**Fig. 4:**
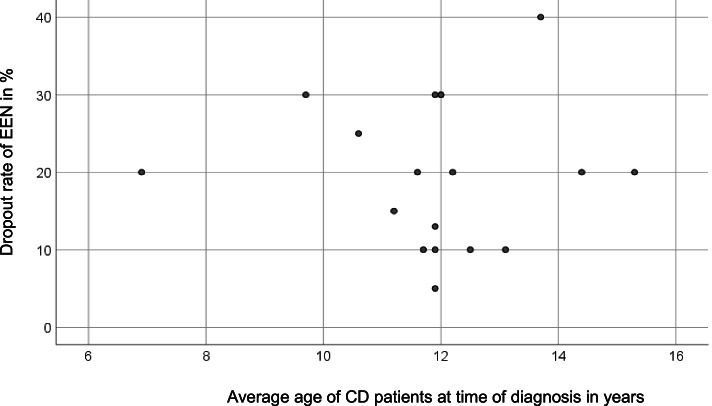
Association between the dropout rate of EEN and the average age of CD patients at the time of diagnosis; Pearson’s correlation; *p* > 0.05; *N* = 18. EEN, exclusive enteral nutrition (own illustration)

22.1% of the CD patients dropped out prematurely. There is no association between the dropout rate of EEN, and the average age of CD patients at the time of diagnosis (*r* = −.01, *p* = .968, *N* = 18) (Fig. 4).

Regarding the dropout rate, no significant difference between the patient education about EEN by a dietician (mdn = 20, IQR = 15–30, *N* = 20) or by a physician or a nurse specialized on IBD was found (mdn =20, IQR = 12.25–26.25, *N* = 10) (*U* = 98.00, *Z* = −.089, *p* = .929, *r* = .016).

The association between duration of patient education about EEN and dropout rate of EEN did not reach statistical significance; however, the correlation between the two variables is of medium strength (the longer the education, the lower the dropout rate) (rs = −.309, *p* = .096, *N* = 30).

**Fig. 5:**
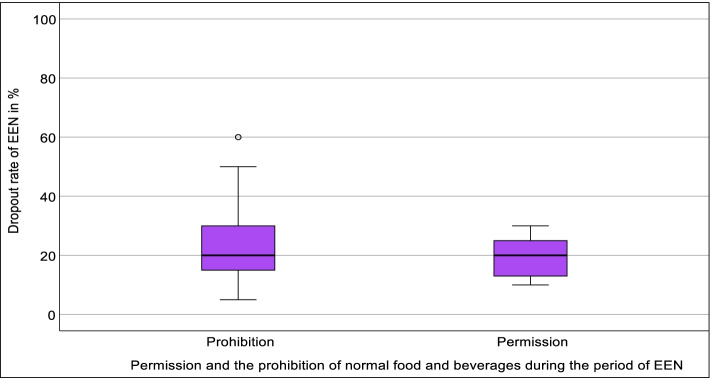
Association between the dropout rate of EEN and permission and prohibition of normal food and beverages during the period of EEN; Mann-Whitney *U* test; *p* > 0.05; *N* = 30. EEN, exclusive enteral nutrition (own illustration)

There was a very small and statistically not significant difference between the permission (mdn = 20, IQR = 12.25–25, *N* = 10) and the prohibition (mdn = 20, IQR = 15–30, *N* = 20) of normal food and beverages during the period of EEN (*U* = 88.50, *Z* = −.514, *p* = 0.607, *r* = 0.09) (Fig. 5).

**Fig. 6:**
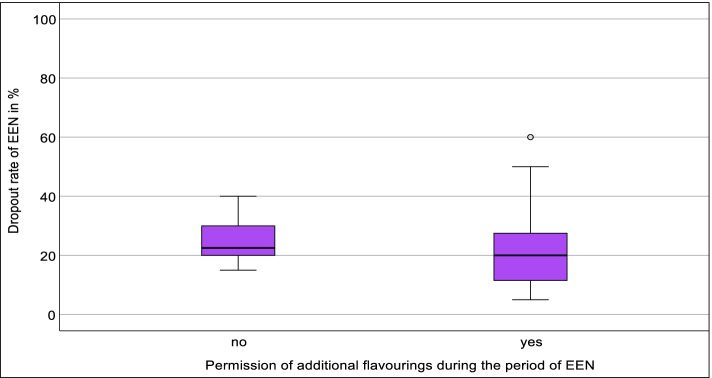
Association between the dropout rate of EEN and permission and prohibition of additional flavorings during the period of EEN; Mann-Whitney *U* test; *p* > 0.05; *N* = 30. EEN, exclusive enteral nutrition (own illustration)

Centers who allowed additional flavorings showed a slightly lower dropout rate, which did not reach statistical significance (mdn = 20, IQR = 11–29, *N* = 24 vs. mdn = 23, IQR = 19–33, *N* = 6) (Fig. 6).

### Process of reintroducing food after EEN

93.5% (29/31) decreased the formula volume over 1 to 5 weeks after EEN. The most common practice was the gradual introduction of food quantity as the formula volume decreased. 6.5% (2/31) recommended an immediate stop of the formula and a rapid start of normal foods and liquids.

83.9% (26/31) recommended a healthy well-balanced diet considering individual intolerances for maintenance therapy. 16.1% (5/31) recommended a light standard diet.

### Supplementary formula after the completion of EEN

34.2% of the CD patients routinely continued PEN after completing the period of EEN (min = 5, max = 90, mdn = 30, *N* = 30). This percentage is the average of the estimated values of the pediatric gastroenterologists, and concrete patient numbers were not requested.

## Discussion

EEN has been established as the gold standard in induction therapy for children and adolescents with CD, but large differences in the administration were observed among the pediatric gastroenterology centers of CEDATA-GPGE®. The variation was observed in the formula used, the application, the addition of flavorings to formula, and foods allowed during the period of EEN as well as the reintroduction of conventional foods after EEN was concluded.

While almost 90% of CD patients at the CEDATA-GPGE® clinics were recommended EEN to induce remission, the use of this dietary intervention varied between 12 and 89% at international studies [8, 9, 11, 14, 15]. 31% of North American medical professionals surveyed reported that they never used an EEN to induce remission, and only 4% prescribed it regularly [10, 14]. The main reason for this was that the medical professionals were concerned about the difficulty in administering EEN and the resulting lack of compliance by the patient and/or family. In addition, the experience of the pediatric gastroenterologists and the frequency in which they used EEN in the past was crucial for the use of this dietary intervention in induction therapy in pediatric CD patients [14, 15]. Our study shows that despite the wide variation in administration and support, EEN is feasible and should be widely used in luminal Crohn’s. The variation in structures and outcomes did not yield clear associations, but should still be used to clarify, compare, standardize, and optimize procedures around EEN.

The international consensus guidelines have recommended that EEN is administered over 6 to 8 weeks [6, 7]. The majority of the German and Austrian practitioners in the current survey utilized EEN over 6 to 8 weeks. In comparison to international practices, a wide difference is evident. Fifty-two percent of Swedish medical professionals recommended a duration of 6 weeks, while 30% of North American medical professionals recommended less than 6 weeks [14, 16]. The majority of Japanese gastroenterologists (76%) prescribed EEN until the person’s symptoms improved (15.9 days on average) [9].

Our study also showed that duration does not seem to influence dropout rate as badly as previously thought, but is more a question of intensive support and reassurance.

Various types of enteral formula can be used for EEN. English and Canadian doctors preferred administration via a nasogastric tube, while the German and Austrian pediatric gastroenterologists of CEDATA-GPGE® and Spanish doctors preferred an oral intake of the formula [14, 15, 17]. These differences may be due to the formula. While the CEDATA-GPGE® clinics and the hospitals in Spain mainly used polymeric formulas for EEN, the physicians from North America, Canada, and Japan mainly used elemental formulas [9, 14, 15]. Due to the poor taste and the associated lower acceptance by the patients, elementary diets usually require application via a gastric tube [18]. According to the S3 and ECCO/ESPGHAN guidelines, low-molecular diets show no advantages in terms of effectiveness compared to high-molecular formula and should only be used in the presence of concomitant diseases, such as a cow’s milk protein allergy [6, 7, 19]. Polymer diets should be preferred to elementary diets [7].

A completion of EEN, which lasts several weeks, is a great challenge for pediatric CD patients and their families. This very monotonous and restrictive form of nutrition, the avoidance of conventional food and drinks, and the taste of the formula often lead to non-compliance and thus to premature termination of EEN [20–22]. In the S3 guideline and in a systematic review by Narula et al., a dropout rate of 20% was reported [21, 22]. The surveyed CEDATA-GPGE® physicians also stated that around a fifth of CD patients discontinued this dietary intervention prematurely. Therefore, the patient’s motivation, close care, and regular follow-ups during the period of EEN are crucial for the full implementation and thus successful treatment of EEN [12, 15].

Although there is no recommendation in the international guidelines regarding flavoring, four-fifths of the CEDATA-GPGE® clinics surveyed allowed the use of flavoring additives during EEN. In international studies, 50 to 81% of gastroenterologists allowed the addition of flavorings to the formula [8, 11, 15, 16]. The intention of medical professionals’ efforts is to avoid taste fatigue or refusal of taste and thus a premature termination of EEN [8].

For the same reasons, a third of the surveyed CEDATA-GPGE® clinics allowed certain conventional foods to be consumed during EEN. In the surveys by Navas-Lopez and Grafors, 9.3 to 81% of gastroenterologists, respectively, allowed the consumption of predefined normal foods and beverages [15, 16].

Although the total exclusion of conventional food and beverages during EEN appears to be crucial for the success of induction therapy for pediatric CD, new modalities of dietary treatment are increasingly being investigated for the nutritional management of CD [23]. For example, the CDED, a food-based diet coupled with PEN, was associated with stable remission rates and a reduction in fecal inflammation levels in pediatric patients with CD [13, 24, 25]. Nevertheless, the CED Working Group of ESPGHAN and the GPGE opposed CDED in the induction therapy of pediatric MC, and thus, the consumption of conventional food and beverages during EEN due to the lack of randomized controlled trials [26, 27]. Further clinical studies are needed to investigate the more tolerable food-based diets—especially regarding the mucosa healing [1].

Regarding the dropout rate, there was no statistically significant correlation between the CEDATA-GPGE® clinics with a short (< 45 min) and long (≥ 45 min) patient education about EEN. However, a tendency was observed in which longer patient’s education about EEN was associated with a lower dropout rate. Comparing the three specialists, pediatric gastroenterologist, IBD nurse, and dietician, it was also observed that a dietician tended to spend more time on the patient education about EEN. This suggests that a dietician should take over the patient information about EEN for pediatric CD patients or that the practitioner should ensure extensive and repetitive advice and support during the process.

The fact that a longer explanation of EEN tended to be associated with a lower dropout rate could be explained by the fact that the professional was able to devote more time to the affected person in a longer consultation or to respond more to them and their needs and wishes. Worries that lead to premature termination of EEN can be reduced during the educational discussion.

There is also no significant correlation between the dropout rate of EEN and the average age of CD patients at diagnosis. Since a premature dropout of the patients is not recorded at the registry, the estimated values of the doctors were used here. To find significant associations, the dropout rates should be included within the documentation sheet of CEDATA-GPGE® in the future.

After EEN, a period of 2 to 3 weeks is recommended for reintroducing conventional foods, with a gradual reduction in formula every 2 to 3 days [7, 19]. There are also considerable differences internationally. While almost 90% of the CEDATA-GPGE® hospitals reduced the formula for 1 to 2 weeks, this period varied between 1 and 12 weeks in European, North American, and Asia-Pacific clinics [11, 15]. In the studies by Stewart and Ho et al., 57 to 76% of North American, Australian, and New Zealand medical professionals allowed one normal meal at a time [8, 14]. Others were guided by the fiber and/or allergen content of the food when reintroducing conventional diets [8, 11, 14].

After the period of EEN, no special diets were recommended to the patients of the clinics of CEDATA-GPGE® for the maintenance therapy. Almost four-fifths of the clinics surveyed recommended a balanced mixed diet, taking individual aversions and intolerances into account. In the studies by Whitten and Ho et al. 45 to 50% of the physicians recommended starting with a low-fiber diet and 17% with a low-allergen diet after the end of EEN [8, 11].

Currently, there is no special “Crohn’s diet” that equally relieves symptoms in all pediatric CD patients [3, 6, 28, 29]. Instead, as part of nutritional therapy with an experienced dietician, the individual diet suitable for the patient should be found on the basis of the severity of the illness, drug treatment, surgical measures, and its accompanying symptoms (e.g., stenosis, short bowel syndrome, stoma) [30].

One of the strengths of this study is the selected data collection technique. A written survey, compared to an interview, is characterized by a fast processing time for the participants. The high response rate of this study should also be emphasized. Postal surveys are usually associated with high non-response rates, which is why this survey clearly stands out with a response rate of 81.6% [31] compared to similar surveys with response rates between 42 and 54% [8, 11].

A major weakness of this study is the small sample size of centers questioned (*N* = 31). In order to identify significant correlations the study should be repeated on a larger scale for multifactorial models and association with outcome parameters.

Another weakness of the survey is the estimated values of the pediatric gastroenterologists for the number of patients who got recommended, started, and stopped EEN prematurely. Specific numbers of patients are missing and should be reevaluated. All data obtained is based on a subjective assessment by the physicians and are therefore inevitably subject to possible bias.

Also to be mentioned is the missing data regarding the PEN (recommended daily calories and how long patients continue to use PEN after EEN) and the formula used by the clinics (e.g., do all centers offer EEN orally first or if the patient struggles with oral formula, do centers switch to different formula type first, or do they immediately escalate to nasogastric tube).

In conclusion, the current study described the wide variation in the attitudes and practice of the German and Austrian pediatric gastroenterologists of the IBD register CEDATA-GPGE® toward the use of EEN in children and adolescents with CD. An active participation of patient registers and further studies is needed to compare existing strategies and to develop consistent approaches to this therapy. Furthermore, on the basis of clinical trials, nutritional interventions should be optimized for induction and maintenance therapy of pediatric CD—including acceptance by the patients and families.

## Supplementary Information

**Additional file 1.** Implementation of EEN in CD_submit_MACP.

## Data Availability

The datasets used and/or analyzed during the current study are available from the corresponding author on reasonable request.

## Abbreviations

CD: Crohn’s disease
CEDATA-GPGE®: Registry for Pediatric Inflammatory Bowel Disease of the Society for Pediatric Gastroenterology and Nutrition
CDED: Crohn’s Disease Exclusion Diet
EEN: Exclusive enteral nutrition
IBD: Inflammatory bowel disease
PEN: Partial enteral nutrition
