# Supplementary material for: Implementation of exclusive enteral nutrition in pediatric patients with Crohn’s disease—results of a survey of CEDATA-GPGE reporting centers
Source: Mol Cell Pediatr. 2022 Apr 5;9:6. doi: 10.1186/s40348-022-00139-x (PMC8982684; doi:10.1186/s40348-022-00139-x)
Supplement: Supplementary file 1 — Additional file 1. Implementation of EEN in CD_submit_MACP. [file 40348_2022_139_MOESM1_ESM.docx]

CEDATA-GPGE® clinic address and outpatient number

**Questions for the doctor**  name

1. How many pediatric Crohn's disease patients do you have per year?

Patients per year

1. Which formula do you predominately use for exclusive enteral nutrition?
   1. Modulen® IBD (Nestlé)
   2. Alicalm (Nutricia)
   3. another formula, which?

1. Which formula do you generally use for exclusive enteral nutrition?

(Multiple answers are possible!)

- 1. Modulen® IBD (Nestlé)
  2. Alicalm (Nutricia)
  3. another formula, which?

1. How long do you recommend an exclusive enteral nutrition?
   1. < 6 weeks
   2. 6 – 8 weeks
   3. > 8 weeks
2. Should you deviate from your basic recommendation regarding the duration of the exclusive enteral nutrition (item 4), what does that depend on?
   1. I deviate from my standard recommendation if / because …

- 1. I recommend basically the same duration for exclusive enteral nutrition.

1. Is your patient (or in the case of children, their parents / guardians) routinely instructed by a nutritionist about the exclusive enteral nutrition?
   1. Yes.
   2. No.
2. Do you usually have a nutritionist who is familiar with the dietary intervention of pediatric Crohn’s disease patients?
   1. Yes.
   2. No.
3. Is the patient generally informed on an outpatient basis or is the start of the exclusive enteral nutrition therapy routinely as part of an inpatient stay?
   1. Outpatient basis.
   2. Inpatient stay.
4. Do you use specific measures to motivate the patient to stick to the diet?

(Multiple answers are possible!)

- 1. Yes, we call the patient or, in the case of younger children, his or her parents / guardians during the exclusive enteral nutrition. How often are these calls made and by whom?

- 1. Yes we do …

- 1. No.

1. What percentage of your patients (estimate) would you recommend exclusive enteral nutrition?

%

1. What percentage of your patients (estimate) start an exclusive enteral nutrition?

%

1. How high is the dropout rate (estimate) of exclusive enteral nutrition?

%

1. What percentage of your patients (estimate) have an oral administration and an application via a nasogastric tube?
   1. % oral administration.
   2. % application via a nasogastric tube.
2. Do you have calprotectin monitoring during exclusive enteral nutrition, for example after three weeks?
   1. Yes. After how many weeks? What conclusions do you draw from this?

- 1. No.

1. Do you usually have a nutritionist who is familiar with the dietary maintenance therapy of pediatric Crohn’s disease patients?
   1. Yes.
   2. No.
2. What percentage of your patients (estimate) continue with a partial enteral nutrition after the end of exclusive enteral nutrition?

%

*Dear doctor!*

*If you answered “yes” to question 7, that means you have a nutritionist in-house who is familiar with pediatric dietary therapy for Crohn's disease patients, please pass the questionnaire on to them for further answers. If you answered the question with "no" and therefore do not have a nutritionist, we would kindly ask you to answer the next questions as well.*

*Thank you very much!*

**Questions for the nutritionist / doctor** name

1. Which dosage do you normally use for exclusive enteral nutrition?
   1. normocaloric (energy density of 1.0 kcal / ml)
   2. highcaloric (energy density of 1.25 – 1.5 kcal / ml)
   3. another dosage, which? What does that depend on?

1. How much time does it take on average to educate the patient (or in the case of children, their parents / guardians) about exclusive enteral nutrition?
   1. < 15 minutes
   2. 15 – < 30 minutes
   3. 30 – < 45 minutes
   4. 45 – < 60 minutes
   5. > 60 minutes
2. Do you gradually introduced exclusive enteral nutrition?

(Multiple answers are possible!)

- 1. Yes, how?

- 1. No.
  2. Different, depending on the patient. What does that depend on?

1. Do you recommend a different preparation for the formula?
   1. No, the formula should only be consumed with water.
   2. Yes, e.g. as ice cream, hot beverage or …?

1. Do you recommend the addition of flavourings to the formula?
   1. Yes, which?

- 1. No, the patient should consume the formula in its pure form, if possible.

1. Do you permit regular food or beverages during exclusive enteral nutrition?

(Multiple answers are possible!)

1. Usually, yes. What do you recommend?

1. Only in exceptional cases. Which foods do you recommend and what are "exceptional cases"?

1. No, the patient should not consume the formula in conjunction with regular food and beverages.
2. How much time does it take on average to educate the patient or, in the case of younger children, the parents about the maintenance therapy nutrition?
   1. < 15 minutes
   2. 15 – < 30 minutes
   3. 30 – < 45 minutes
   4. 45 – < 60 minutes
   5. > 60 minutes
3. Do you gradually reduce the formula after the period of exclusive enteral nutrition?
   1. No, the patient could stop the formula immediately and start with normal food and beverages.
   2. Yes, we reduce the formula gradually. How?

1. Do you recommend a special diet for the maintenance therapy?
